# Screening for cognitive impairment among patients with work-related stress complaints in Denmark: validation and evaluation of objective and self-report tools

**DOI:** 10.5271/sjweh.3990

**Published:** 2021-12-30

**Authors:** Johan Høy Jensen, Kamilla Woznica Miskowiak, Scot E Purdon, Jane Frølund Thomsen, Nanna Hurwitz Eller

**Affiliations:** 1 Department of Occupational and Environmental Medicine, Bispebjerg Hospital, Copenhagen, Denmark; 2 Copenhagen Stress Research Center, Copenhagen, Denmark; 3 Psychiatric Centre Copenhagen, Rigshospitalet, Copenhagen, Denmark; 4 Department of Psychology, University of Copenhagen, Copenhagen, Denmark; 5 Department of Psychiatry, University of Alberta, Edmonton, AB, Canada; 6 Department of Neuropsychology, Alberta Hospital Edmonton, Edmonton, AB, Canada

**Keywords:** assessment, attention, burnout, exhaustion disorder, memory, neuropsychological test, SCIP, self-rated health, working memory

## Abstract

**Objective:**

Many patients with work-related stress display cognitive impairment that may hamper recovery. We examined objective and subjective tools for screening of cognitive impairment in this patient group.

**Methods:**

Patients were assessed with Danish versions of the objective Screen for Cognitive Impairment in Psychiatry (SCIP-D), standardized neuropsychological tests that tapped into the same cognitive domains, the self-assessed Cognitive Failure Questionnaire (CFQ), and several additional scales of symptom severity and psychosocial status. Concurrent validity of the SCIP-D and CFQ was assessed by calculation of Pearson’s correlation coefficients between the objective and subjective tools and the scores on more conventional standardized neuropsychological tests. Decision validity was assessed with logistic receiver-operating-characteristic analyses using a cut-score approach to the objective and the subjective test results to predict impairment detected by the standardized tests. Cognitive norms were established through the data of 79 healthy controls. SCIP-D scores were compared between patients and healthy controls with independent t-tests.

**Results:**

We included 82 patients with work-related stress. The SCIP-D total scores were strongly associated with standardized neuropsychological tests (r=0.76, P<0.001). The self-assessed CFQ was not associated with either measure of objective cognitive functioning (r≤0.12, P≥0.30). The optimal SCIP-D total-score cut of ≤72 identified 43.2% of the patients with global objective cognitive impairment. The patients performed mildly-to.moderately lower than the healthy controls on the SCIP-D total score (Cohen’s d=0.39) and the subtests for working memory (d=0.39) and processing speed (d=0.61).

**Conclusion:**

The SCIP-D is a valid screening tool sensitive to objective performance-based cognitive impairment among patients with work-related stress.

Prolonged work-related stress has vast personal, economic, and societal costs as it may impede functional and work capacity (1). In Nordic countries and The Netherlands, occupational clinics manage work-related stress to promote recovery and labor market attachment. Many patients exposed to long-term stress report memory and concentration difficulties (ie, cognitive impairment) as a core feature in addition to symptoms of depression, anxiety, fatigue, sleep problems, and social withdrawal (2, 3). As most modern jobs require complex cognitive skills, it is likely that consideration of cognitive impairment in clinical management of work-related stress may improve occupational recovery, eg, when discussing strategies for cognitive remediation, adjustments of job tasks, and the optimal time for return to work (4–7).

Since the mid-2000s, there have been several reports of mildly-to-moderately impaired performance on neuro­psychological tests for verbal learning and memory, executive functioning, processing speed, and attention in patients with work-related stress (2, 8–10). Longitudinal studies suggest that subjectively self-reported and objectively measured cognitive impairments may persist several years after seeking healthcare support (10, 11). Indeed, growing evidence indicates no or weak association between subjective and objective measures of cognitive impairment in this patient group (8, 12, 13). A recent systematic review on age-related cognitive decline concluded that studies employing more comprehensive self-report measures of cognitive impairment were more likely to detect objective cognitive impairment (14).

In Danish occupational clinics, neuropsychological functioning is not routinely examined in patients with work-related stress, as such assessment requires resource-consuming procedures conducted by specialized staff. Considering the potential value of improved efforts to enhance recovery, there is a need for optimizing systematic assessment of cognitive impairment by virtue of a brief and inexpensive screening tool suitable for reliable administration after a relatively brief period of professional training.

The Screen for Cognitive Impairment in Psychiatry (SCIP) is a feasible objective cognitive screener (15) that has been validated with good psychometric properties among psychiatric populations in several languages (15–17), including Danish (SCIP-D) (18, 19). The SCIP-D exists in three parallel versions for longitudinal monitoring and consists of five subtests assessing verbal memory, working memory/executive skills, and visuomotor processing speed. The subscale raw scores can be summed to provide a total score to quickly offer an index of global cognitive impairment. Given the symptomatic similarities between patients with work-related stress and affective disorders, the SCIP-D is a promising tool to screen for cognitive impairment in several cognitive domains relevant to patients with work-related stress.

Further, the 25-item self-report Cognitive Failure Questionnaire (CFQ) is a comprehensive well-validated global trait measure of subjective cognitive difficulties in daily life (eg, at work) covering deficits in memory, planning, forgetting names, attention, and motor function (20). The CFQ has shown only weak correlations with social desirability and neuroticism, but strong associations with psychological strain (20, 21). Patients with work-related stress have previously reported higher CFQ total scores [mean 54.4, standard deviation (SD) 14.1] than healthy controls (HC) (mean 24.9, SD 10.8) (2), and there have been preliminary reports of associations with attentional difficulties in individuals suffering from job burnout (22). The CFQ may correctly identify actual global cognitive deficits given the comprehensive scope of the scale (14).

The current study aims were to (i) assess the concurrent validity of the SCIP-D and the CFQ through associations with neuropsychological functioning in a sample of patients with work-related stress complaints, (ii) determine the optimal cut-off scores on the SCIP-D and on the CFQ for prediction of objective cognitive impairment quantified by a battery of standardized neuropsychological tests, (iii) assess the sensitivity of the SCIP-D to cognitive impairment in patients with work-related stress through comparisons with HC, and (iv) investigate the equivalence of the three parallel versions of the SCIP-D within the patient sample.

## Methods

### Patients and procedures

This cross-sectional study included adult outpatients with work-related stress (ICD-10 codes F43.2; F43.9; Z56) recruited consecutively from March 2019 through February 2020 in the Stress Reduction Clinic at the Department of Occupational and Environmental Medicine, Bispebjerg Hospital. The inclusion criteria for the patients comprised 18–64 years of age, significant work-related exhaustion symptoms (Karolinska Exhaustion Disorder Scale, 9-item, [KEDS]: total scores ≥20) (23, 24), and native Danish language. The exclusion criteria were dyslexia, alcohol or substance abuse, substantial somatic illness, somatic illness or disability known to cause cognitive impairment, personal history of clinical depression, and current psychiatric illness; however, we allowed for mild depressive symptoms defined as Hamilton Depression Rating Scale, 6-item, (HDRS-6) (25) scores ≤8.

Initially, patients referred to the department for examination of work-related stress were screened for study eligibility by medical doctors. For research purposes, eligible patients attended a single 1.5-hour session in the clinic administered by the first author for obtaining background information, assessment of neuropsychological functioning, completion of self-reported cognitive impairment, and rating of depressive symptoms.

All participants provided informed written consent and were offered a small gift card certificate for their study participation. According to the local ethics committee, ethical approval was not required as the study did not involve biomedical or invasive procedures. The study complies with the Helsinki Declaration of 1964 and its later amendments.

### Background information

Patients’ data on background information included age, sex, years of education, premorbid verbal intelligence [Danish Adult Reading Test (DART)] (26, 27), occupational status, marital status, number of days sick-listed, and number of previous work-related stress episodes, depressive symptoms (25), non-restorative sleep (28), and perceived stress (29, 30). Patients without sleeping disturbances per se (eg, sleep onset, interruptions) may still feel unrefreshed upon awakening. Non-restorative sleep within the past seven days was assessed by the 9-item Restorative Sleep Questionnaire Weekly Version (RSQW) using a five-point Likert scale (ranging from 1=“Not at all” to 5=“Completely”) (supplementary material, www.sjweh.fi/article/3990, item 1) with lower scores indicating a worse non-restorative sleep. The scale was computed by the following formula (28):

Premorbid verbal intelligence was estimated by the following formula (26):

### Objective measures of cognitive status

All patients were assessed with SCIP-D form 3, while the SCIP-D forms 1 and 2 were administered alternately between patients by the end of each session. The SCIP-D has five subtests and the instrument is feasible to administer (<20 minutes) by trained staff for assessment of verbal learning and memory (VLT-I), delayed memory (VLT-D), working memory (WMT), verbal fluency (VFT), and processing speed (PST). For administration details see (15). Each SCIP-D subtest was scored by summing correct responses to that test (eg, no correct responses would score 0, which is the lowest score possible). The SCIP-D total-score index of global cognitive functioning was computed by summing the raw scores of all five subtests. The VFT (and hence the SCIP-D total score) has no upper limit score.

Consistent with previous studies validating the SCIP-D (18, 19), we matched the five SCIP-D subtests to corresponding standardized neuropsychological tests tapping into the same cognitive domains: Rey Auditory Verbal Learning Test (RAVLT) total recall across the five learning trials (I-V) (31) (SCIP-D: VLT-I), the Repeatable Battery for the Assessment of Neuropsychological Status (RBANS) Digit Span Forward (32), the WAIS-III Letter-Number Sequencing (33) (SCIP-D: WMT), verbal fluency tests with the letters S and D (34) (SCIP-D: VFT), RAVLT recall following interference and 30 min. delay (SCIP-D: VLT-D), and the Trail Making Test part A (35) (SCIP-D: PST). Multiple neuropsychological tests were matched to reflect the cognitive functions assessed by the SCIP-D total score and subtests for WMT and VLT-D, respectively (table 1).

**Table 1:** Danish version of the Screen for Cognitive Impairment in Psychiatry (SCIP-D) total score and subtests matched according to the corresponding measures of standardized neuropsychological tests loading on similar cognitive domains (vertical). Screening tool scale. [RAVLT=Rey Auditory Verbal Learning Test; RBANS=Repeatable Battery for the Assessment of Neuropsychological Status; WAIS-III=Weschler’s Adult Intelligence Scale, third version].

	Standardized neuropsychological measure	Cognitive domain
SCIP-D total score	Mean composite score for the cognitive domains: memory, executive skills, and visuomotor processing speed	Global cognitive functioning
Verbal Learning Test, Immediate	RAVLT total recall across the five learning trials (I–V)	Immediate memory
Working Memory Test	Mean composite score for RBANS Digit Span Forward and WAIS-III Letter-Number Sequencing	Working memory/ executive skills
Verbal Fluency Test	Verbal fluency with the letters S and D	Verbal fluency/ executive skills
Verbal Learning Test, Delayed	Mean composite score for RAVLT recall following interference (trial VI) and RAVLT recall following 30 minutes delay	Delayed memory
Processing Speed Test	Trail Making Test part A ^a^	Processing speed
Cognitive Failure Questionnaire total score	Mean composite score for the cognitive domains: memory, executive skills, and visuomotor processing speed	Global cognitive functioning

a Scores inversed to adjust for negative proportionality.

### Healthy control (HC) sample

A patient-matched HC sample was created to establish robust thresholds for identification of cognitive impairment (yes/no). We used bootstrapping with 1000 repeatedly samplings to match a pre-existing data pool of HC (N=103) (19) to the patient sample (N=82) according to stratas of sex, age, verbal intelligence (tertiles) (36). This technique matches each patient to 1000 randomly drawn HC (with replacement) from the data pool having the same combination of stratas (eg, middle-aged female with a high premorbid intelligence). This implies that the same individual HC could be matched to multiple patients and that multiple HC could be matched to the same individual patient. We chose this approach to introduce novel norm variation, because the HC were recruited for a previous study validating the SCIP-D in psychiatric populations (18, 19).

The 103 HC were recruited in the blood bank at Copenhagen University Hospital, Rigshospitalet from January 2014 to June 2015 and assessed with identical tests administered in the same order as the current patients. Background information of the HC included age, sex, verbal intelligence (26, 27), perceived stress (29, 30), and the Hamilton Depression Rating Scale, 17-item version (25). For details see (19). In this study, we applied an additional HC exclusion criterion of excess perceived stress defined as a score of ≥20 on the Perceived Stress Scale corresponding to ≥1 SD above the general population mean (29, 30).

### Normative objective cognitive functioning

All neuropsychological test scores of the patients were z-score standardized to the matched HC sample (mean 0, SD 1) allowing immediate comparison with normative scores. The z-scores for Trail Making Test part A were inversed to adjust for negative proportionality. The mean composite scores were computed by averaging the z-scores of the subscales (table 1).

### Subjective measure of cognitive status

Subjective cognitive functioning was assessed by the 25-item Cognitive Failure Questionnaire (CFQ) using a five-point Likert scale (ranging from 0=“Never” to 4=“Very often”) (20). The CFQ total score was computed by summing the rating scores for all 25 items (scale: 0–100). A higher CFQ total score indicates more lapses of attention, perception and memory in daily life, eg, losing task goals during execution and forgetting names more often (21). The CFQ total score was matched to the mean composite measure for global cognitive functioning to tap into the same cognitive domains (table 1). There were no CFQ norm data in the matched HC sample.

### Statistical analyses

All statistical analyses were performed using SAS Studio version 3.8 (SAS Institute, Cary, NC, USA) on a logged server hosted by the Capital Region of Denmark. The general alpha level of statistical significance was set to 0.05 unless otherwise stated.

The concurrent validity of all SCIP-D scores and the CFQ total score (aim i) was assessed using Pearson’s correlation coefficient according to their matched standardized neuropsychological measure. As a sensitivity analysis, we examined the adjusted association between objective and subjective measures of cognitive status using linear regression to evaluate the potential bias from covariation by age, sex, years of education, premorbid verbal intelligence, occupational status, marital status, days sick-listed, previous episodes, depressive symptoms, non-restorative sleep, and perceived stress.

We determined the clinical applicability of SCIP-D and CFQ for correctly discriminating between true cases/non-cases of objective cognitive impairment (aim ii). This was conducted using logistic receiver-operating-characteristic (ROC) regression analyses for all SCIP-D scores and the CFQ total score according to objective cognitive impairment classified by each of their matched neuropsychological measures (yes/no) (37). Adding to the clinical applicability of the results, we computed a conservative and a relaxed set of thres­holds indicating objective cognitive impairment: the conservative thresholds were 1.5 SD and 2.0 SD below the HC mean z-scores for global and focal cognitive impairment, respectively, while the relaxed thresholds were 1.0 SD and 1.5 SD below each of the same HC measures of cognition, respectively.

We compared differences in SCIP-D total scores and subtest scores between patients and HC (aim iii) using independent t-test (two-tailed). Cohen’s d values were calculated to determine the effect sizes. For differences in the five SCIP-D subtest scores, the threshold for statistical significance was set to an alpha level of 0.01 to adjust for multiple comparisons.

Finally, we assessed concurrent validity of the SCIP-D forms 1-3 (aim iv) by analyzing Person’s correlation coefficients between the total scores of all three parallel SCIP-D versions and the standardized measure of global cognitive functioning.

## Results

A total of 369 patients were referred to the clinic of whom 110 were eligible for this study. Of these, 28 patients were excluded for the following reasons: no wish to participate (N=10), no response to attempted contact via telephone (N=12), displayed excess depressive symptoms (N=4), and other reasons (N=2). Consequently, 82 patients attended a 1.5-hour assessment session defining the patient sample (supplementary material 2). Of these, 12 patients did not complete one of the alternated SCIP-D forms 1-2 due to excess fatigue ending the assessment session.

In the bootstrapping procedure for establishment of the matched HC sample, we excluded four female patients due to missing data or no available HC match according to the three matching variables. We excluded one HC from the total data pool due to excess perceived stress. Thus, we were able to match a total 78 patients to randomly drawn data from 79 individual HC. Finally, the matched HC sample included 78 000 HC observations as we selected 1000 bootstrapping resamples to the 78 patients eligible for matching (table 2). We assigned the HC norms scores to the full patient sample (N=82).

**Table 2:** Establishing the matched healthy control (HC) sample to patients according to verbal intelligence, age (tertiles), and sex.

Matching variables			Number of patients (N=82)		Number of matchable HC according to patients (N=79)		HC sample ^a^ (N=78,000)	
			
Verbal intelligence ^b^	Age ^c^	Sex	N^d^	% of N	N^d^	% of N	N	% of N
“missing”, 1, 2	1, 2	F	4	4.9	0	0.0	0	0.0
1	1	F	12	14.6	12	15.2	12 000	15.4
1	1	M	≤3	≤3.7	12	15.2	≤3000	≤3.9
1	2	F	9	11.0	≤3	≤3.9	9000	11.5
1	2	M	≤3	≤3.7	≤3	≤3.9	≤3000	≤3.9
1	3	F	≤3	≤3.7	≤3	≤3.9	≤3000	≤3.9
2	1	F	9	11.0	11	13.9	9000	11.5
2	2	F	9	11.0	≤3	≤3.9	9000	11.5
2	3	F	10	12.2	4	5.1	10 000	12.8
3	1	F	≤3	≤3.7	14	17.7	≤3000	≤3.9
3	2	F	7	8.5	9	11.4	7000	9.0
3	2	M	≤3	≤3.7	≤3	≤3.9	≤3000	≤3.9
3	3	F	8	9.8	5	6.3	8000	10.3
3	3	M	4	4.9	≤3	≤3.9	4000	5.1

a The HC sample (N=78,000) was established based on randomly drawn data from 79 individual HC matched to 78 individual patients with complete data according to verbal intelligence, age (tertiles), and sex using bootstrapping with 1000 resamples.

b 1=101.4–111.4; 2=111.4–114.7; 3=114.7–126.3

c 1=19–40; 2=40–52; 3=52–63.

d For anonymization reasons, we collapsed the matching variable values for patients with no HC match (N=4) and set cells with few observations to ≤3.

Table 3 presents the characteristics of the patient sample and the matched HC sample (N=78 000). The patient and HC samples were comparable regarding the sex composition, years of age, and estimated verbal intelligence. On average, the patients performed -0.9 SD lower than the HC mean on the global composite score of the standardized neuropsychological tests. The patients rated an average CFQ total score of 52.0 (SD 11.7). Supplementary material 3 presents raw scores of the measures for objective cognitive status.

**Table 3:** Characteristics and demographics of the patient and healthy control (HC) samples. [HC=healthy control; SD standard deviation; CFQ=Cognitive Failure Questionnaire; CI=cognitive impairment].

	Patient sample (N=82)			HC sample (N=78 000)		
	
	Mean	SD	%	Mean	SD	%
Individuals, N	82			79		
Sex						
Females			85			88
Males			15			12
Age	45.7		10.6	42.5	13.1	
Estimated verbal intelligence	113.0	4.5		114.1	5.2	
Years of education	15.4	2.2		15.7	2.9	
Perceived stress scale score	22.2	3.0		6.5	4.3	
Hamilton Depression Rating Scale						
6-items	5.6	1.8				
17-items				1.3	1.6	
Karolinska Exhaustion Disorder Scale	30.5	5.8				
Non-restorative sleep scale	39.3	15.7				
Working/job seeking			7			
Full-time sick-listed			62			
Part-time sick-listed			30			
Days sick-listed (min-max: 3–562)	117.3	101.8				
Previous stress episodes (min-max: 0–2)	0.6	0.7				
Global cognitive functioning						
Composite neuropsychological test score, objective cognition (low-high functioning ^a^	-0.9	1.6		0.0	1.0	
CI (conservative threshold) ^b^			32			4
CI (relaxed threshold) ^b^			45			21
SCIP-D total score, objective cognition (low-high functioning)	74.2	9.7		77.7	8.4	
CFQ total score, subjective cognition (high-low functioning)	52.0	11.7				

a Mean composite z-score standardized to the HC sample (mean 0, SD 1).

b Conservative threshold: 1.5 SD below HC global mean, relaxed threshold: 1.0 SD below HC global mean.

### Concurrent validity (aim i)

We found that all SCIP-D scores were correlated with their matched standardized measure of cognitive functioning (SCIP-D total score: r=0.76, subtest scores: r=0.40-0.70, P<0.001). The CFQ total score was neither associated with the SCIP-D total score (r=-0.01, P=0.96) nor the standardized measure of global cognitive functioning (r=-0.12, P=0.30). Based on visual inspection of scatterplots and Q-Q-plots, correlation coefficients were unsusceptible to bias from outliers or non-linear associations (data not shown). The finding of no correlation between objective and subjective measures of cognitive status was supported when adjusting for covariation by age, sex, years of education, premorbid verbal intelligence, occupational status, marital status, days sick-listed, previous episodes, depressive symptoms, non-restorative sleep, and perceived stress (unadjusted model: beta_CFQ Total Score_=-0.02, P=0.30 versus the adjusted model: beta_CFQ Total Score_>-0.001, P=0.80).

### Optimal cut-offs (aim ii)

Table 4 presents the proposed cut-off values for the SCIP-D total and subtest scores to identify patients as “cognitively impaired” based on conservative (ie, 1.5 SD and 2.0 SD below the HC mean for global and focal cognitive impairment, respectively) and more relaxed impairment thresholds (ie, 1.0 SD and 1.5 SD, respectively). The proposed conservative SCIP-D total-score cut-off of ≤72 classified 43.2% of the patients with cognitive impairment compared to 28.6% of the HC (sensitivity: 0.77, specificity: 0.73). The proposed CFQ cut-off value of ≥54 yielded lower values of sensitivity (0.52) and specificity (0.63), suggesting a poor clinical applicability of the self-assessed CFQ for detection of objective cognitive impairment.

**Table 4:** Proposed cut-off values for the Screen for Cognitive Impairment in Psychiatry, Danish version (SCIP-D) and Cognitive Failure Questionnaire (CFQ) to detect objective cognitive impairment (standardized neuropsychological tests) among patients with work-related stress. [AUC=area under curve].

Test	Cut-off ^a^	Sensitivity	Specificity	AUC	95% CI	Cognitive impairment, %	

						Patients	Healthy controls
SCIP-D total score							
Conservative ^b^	≤72	0.77	0.73	0.84	0.76–0.93	43.2	28.6
Relaxed ^c^	≤75	0.83	0.76	0.85	0.77–0.94	51.0	40.6
Verbal Learning Test, immediate							
Conservative ^b^	≤16	1.00	0.95	0.97	0.93–1.00	7.3	1.9
Relaxed ^c^	≤19	1.00	0.87	0.98	0.94–1.00	19.5	6.7
Working Memory Test							
Conservative ^b^	≤17	0.50	0.76	0.70	0.55–0.85	28.0	16.6
Relaxed ^c^	≤18	0.61	0.71	0.70	0.59–0.82	38.3	30.6
Verbal Fluency Test							
Conservative ^b^	≤11	0.80	0.79	0.87	0.78–0.96	28.0	17.2
Relaxed ^c^	≤13	0.83	0.72	0.87	0.79–0.95	40.2	28.7
Verbal Learning Test, delayed							
Conservative ^b^	≤5	0.63	0.92	0.83	0.64–1.00	13.4	15.1
Relaxed ^c^	≤6	0.60	0.79	0.77	0.63–0.91	28.0	24.1
Processing Speed Test							
Conservative ^b^	≤8	0.50	0.90	0.75	0.64–0.87	21.0	4.1
Relaxed ^c^	≤10	0.69	0.55	0.69	0.56–0.81	53.1	27.2
CFQ total score							
Conservative ^b^	≥54	0.52	0.63	0.55	0.41–0.70	42.0	
Relaxed ^c^	≥54	0.50	0.64	0.55	0.42–0.69	42.0	

a Proposed cut-off values were provided by receiver-operating-characteristic (ROC) analyses using logistic regression.

b Conservative (recommended) thresholds of 1.5 and 2.0 standard deviations (SD) below healthy control (HC) mean scores for global and focal cognitive impairment, respectively.

c Relaxed thresholds of 1.0 and 1.5 SD below HC mean scores for global and focal cognitive impairment, respectively.

### Sensitivity of the SCIP-D to cognitive impairment in patients with work-related stress (aim iii)

On the SCIP-D, the patients scored significantly lower on the total score and the subtests for working memory (Cohen’s d-values=0.39) and visuomotor processing speed (d=0.61) compared to the HC (table 5). We found no differences in the severity of cognitive complaints among patients identified with and without cognitive impairment according to the conservative SCIP-D total-score cut of ≤72 (mean 52.1, SD 12.5 and mean 51.9, SD 11.3, respectively, t(78)=-0.06, P=0.95).

**Table 5:** Differences in the Screen for Cognitive Impairment in Psychiatry, Danish version (SCIP-D) total and subtest scores between the patient and healthy control samples. [VLT-I=verbal learning test, immediate; WMT=working memory test; VFT=verbal fluency test; VLT-D=verbal learning test, delayed; PST=processing speed test; SD standard deviation].

SCIP-D	SCIP-D scores, mean (SD)		t ^a^	df	P-value	Cohen’s d

	Patients	HC				
SCIP-D total score	74.2 (9.7)	77.7 (8.4)	3.75	79079	<0.001	0.39
VLT-I	22.5 (3.3)	23.2 (3.0)	2.30	79080	0.02	0.24
WMT	19.0 (2.7)	19.9 (2.4)	3.75	79080	<0.001	0.39
VFT	14.9 (4.5)	15.5 (4.2)	1.28	79080	0.20	0.14
VLT-D	7.4 (2.0)	7.5 (2.2)	0.32	79080	0.75	0.04
PST	10.4 (2.5)	11.8 (2.1)	5.10	80.119	<0.001	0.61

a Differences in SCIP-D total and subtest scores between the samples were analyzed using independent samples t-test (two-tailed). Significance alpha levels were set to 0.05 and 0.01 for the SCIP-D total score and the subtest scores, respectively.

### Equivalence of the SCIP-D versions (aim iv)

Total scores of the SCIP-D form 3 and the standardized measure of global cognitive functioning were highly correlated with total scores of SCIP-D forms 1 (N=35) and 2 (N=34) (r≥0.65, P<0.001).

## Discussion

We evaluated objective (performance-based) and subjective (self-report) tools for screening of neurocognitive impairment among patients with work-related stress. Associations with comprehensive neuropsychological tests indicated good concurrent validity of the objective cognitive screener, SCIP-D, but not the subjective tool, CFQ. The SCIP-D showed superior decision validity to the CFQ using a cut-score approach for correct classification of patients with objective cognitive impairment. Specifically, the two SCIP-D subtests for working memory and processing speed were particularly sensitive, while the SCIP-D subtests for verbal fluency, learning and memory recall were not statistically different between patients and HC. The equivalence of the three parallel versions of the SCIP-D among patients was indicated.

Corroborating earlier studies, we found no significant association between objective and subjective measures of cognition (8, 12–14, 18, 19). This finding prevailed when adjusting for potential covariates, suggesting that the lack of association was not attributable to bias by confounding, such as depressive symptoms, consistent with previous results (12). As demonstrated in affective disorders, the SCIP-D yielded valid psychometric properties for screening of objective cognitive impairment among patients with work-related stress (18, 19).

A conservative SCIP-D total-score cut of ≤72 yielded marginally lower sensitivity and specificity values among patients than a more relaxed cut of ≤75 did. However, the conservative SCIP-D total-score cut classified 28.6% of HC as cognitively impaired, while this number was 40.6% for the relaxed cut, suggesting an excess false-positive rate in the present HC sample. We identified about half of the patients with mild-to-moderate global objective impairment in line with previous findings among comparable (3, 9) and psychiatric populations (18, 19). Particularly, the present patients displayed lower performance on the subtests for processing speed (Cohen’s d=0.61) and working memory (d=0.39) (38) comparable to point d-estimates of similar patients recruited in another Danish department of occupational medicine (d=0.69, P<0.01 and d=0.28, P=0.13, respectively) (5). We observed no significantly impaired performance on the remaining subtests, although the scores for immediate verbal learning indicated mild memory dysfunctions relative to the HC (P=0.02). This should not be taken as strong evidence for the non-existence of impairments in verbal fluency and delayed recall in this patient group, as prior studies have reported impaired performance in these cognitive functions (8–10, 39). Patients identified with and without global cognitive impairment on the SCIP-D reported similar levels of subjective cognitive impairment, supporting that performance-based and self-report cognitive deficits are uncorrelated features in patients with work-related stress.

The concurrent validity of the parallel SCIP-D forms is in keeping with previous findings (18, 19), suggesting that the three versions could be administered for tracking of cognitive status. Yet, more studies using a randomized administration order in work-related stress are needed to support such applicability of the SCIP-D.

The patients’ average CFQ total score for subjective cognitive impairment concurred with previous findings (mean 54.4, SD 14.1) (2). We had no HC data available on the CFQ measure in the present study. However, according to another similarly composed HC sample with normative CFQ data (mean 24.9, SD 10.8) (2), 82.7% of the current patients can be identified with global subjective cognitive impairment according to a conservative cut-off (ie, CFQ total score ≥41). This suggests that the prevalence of subjective cognitive impairment is higher than objective cognitive impairment in work-related stress and that self-assessment of cognitive status cannot replace objective performance testing of cognitive skills. Moreover, we determined the same optimal CFQ total-score cut with unsatisfactory area-under-curve values according to both impairment thresholds, reflecting the poor decision validity of the CFQ for detection of performance-based cognitive deficits.

In line with recommendations from psychiatry (18, 19), we propose that cognitive impairment is evaluated with a brief objective cognitive screener in addition to subjective cognitive difficulties among patients with work-related stress. If using the SCIP-D for this purpose, we recommend applying the proposed conservative thresholds for cognitive impairment (eg, SCIP-D total score ≤72), since the present results suggested a higher false-positive rate – at least among the HC sample. In addition, we advocate that the SCIP-D cut scores are interpreted in accordance with clinical judgement to individually account for premorbid factors linked to cognitive functioning, eg, age, years of education, estimated premorbid intelligence (40). The brevity of the SCIP-D for feasible screening of impairment is a trade-off for a more in-depth insight into neurocognitive functioning. Therefore, the SCIP-D should only be administered for screening purposes and not replace a full-scale neurocognitive examination.

Ellbin et al (8) previously validated the Swedish version of the Cognitive Assessment Battery as an objective cognitive screener in stress-related exhaustion. The CAB comprises six subtests assessing similar cognitive domains as the SCIP-D, although the CAB has no measure for global cognitive status. To our knowledge, the CAB only exists in one form poorly applicable to longitudinal monitoring of cognitive status due to learning bias.

The current study has some limitations. The proportion of patients identified with cognitive impairments may be underestimated due to few severely affected patients in the sample. Patients with greater exhaustion symptoms and/or cognitive impairment may lack the vigor and motivation to volunteer for a demanding assessment session. More severely affected patients may display greater subsidiary depressive symptoms of HDRS-6 scores >8, which was an exclusion criterion for study participation. The HC sample size was insufficient to calculate demographically adjusted norm cut-offs for cognitive impairment (40) in contrast to a more simple “one-cut” approach based on average scores in the present study. The normative sample was established based on a data pool of HC recruited in a previous study (18, 19), which potentially replicates systematic error, if any. This source of error was reduced as we matched eligible HC using a bootstrapping technique that introduced unique variation in normative cognitive functioning. Finally, the present study did not include a measure of insufficient effort (eg, Test of Memory Malingering, [TOMM]) (41). It is possible that some test takers may intentionally underperform, though this seems unlikely as participation was voluntary. Our clinical experience is that the vast majority of occupational patients with prolonged work-related stress wish to resume their normal work life.

Strengths of the study were the large patient sample (N=82) matched with data from 79 individual HC, providing statistical power to evaluate the study aims. It was also a strength that we validated a feasible objective cognitive screener with parallel forms that can be implemented in occupational clinics and research for administration by non-specialist healthcare providers with some experience in assessment. Specifically, in addition to subtest scores for five typically affected cognitive functions, the SCIP-D offers a total score that is easy to interpret and evaluate. Administration of the SCIP-D is brief, which does not fatigue the patients as much as a conventional neurocognitive examination.

### Concluding remarks

In this study, patients with work-related stress showed impaired performance on tests for global cognitive functioning, particularly processing speed and working memory, while objective and subjective measures of cognitive status were poorly correlated. The objective cognitive screener, SCIP-D, was a valid and feasible tool to identify and monitor objective cognitive impairment using a cut-off score in this patient group. Based on these findings, we recommend screening for objective cognitive impairment using the Danish SCIP-D evaluated according to the conservative cut-off scores and clinical judgment among patients with work-related stress, who complain about cognitive difficulties.

## Supplementary material

Supplementary material
