# Supplementary material for: Screening for cognitive impairment among patients with work-related stress complaints in Denmark: validation and evaluation of objective and self-report tools
Source: Scand J Work Environ Health. 2021 Dec 30;48(1):71–80. doi: 10.5271/sjweh.3990 (PMC8729168; doi:10.5271/sjweh.3990)
Supplement: Supplementary material [file SJWEH-48-71-S001.pdf]

Screening for cognitive impairment among patients with work-related stress complaints in Denmark: validation and evaluation of objective and self-report tools[1]

by Johan Høy Jensen, PhD,[2] Kamilla Woznica Miskowiak, DMSc, Scot E Purdon, PhD, Jane Frølund Thomsen, PhD, Nanna Hurwitz Eller, DMSc

1. *Supplementary materials*
2. *Correspondence to: Johan Høy Jensen, Department of Occupational and Environmental Medicine, Bispebjerg Hospital, Bispebjerg Bakke 23F, DK-2400, Copenhagen NV, Denmark. [E-mail: johan.hoey.jensen@regionh.dk]*

Supplementary material 1. The Restorative Sleep Questionnaire Weekly Version (RSQW).

From: Drake, C. L., Hays, R. D., Morlock, R., Wang, F., Shikiar, R., Frank, L., ... & Roth, T. (2014). Development and evaluation of a measure to assess restorative sleep. *Journal of Clinical Sleep Medicine*, *10*(7), 733-741.
The following questions ask about how you felt when you woke up and started the day during the past 7 DAYS.
When answering the questions think of how you felt about thirty minutes after getting out of bed to start the day. For each question below, please circle the number that best indicates how you feel (circle only one number for each question).

To what extent do you feel… (rating ranged from 1=”Not at All” to 5=”Completely”): 1. Tired? (R) 2. Sleepy? (R) 3. In a good mood? 4. Rested? 5. Refreshed or restored? 6. Ready to start the day? 7. Energic? 8. Mentally alert? 9. Grouchy?, where R= reverse scoring.

Supplementary material 2. Flow of patients for study participation. HDRS-6 = Hamilton Depression Rating Scale, 6 items.

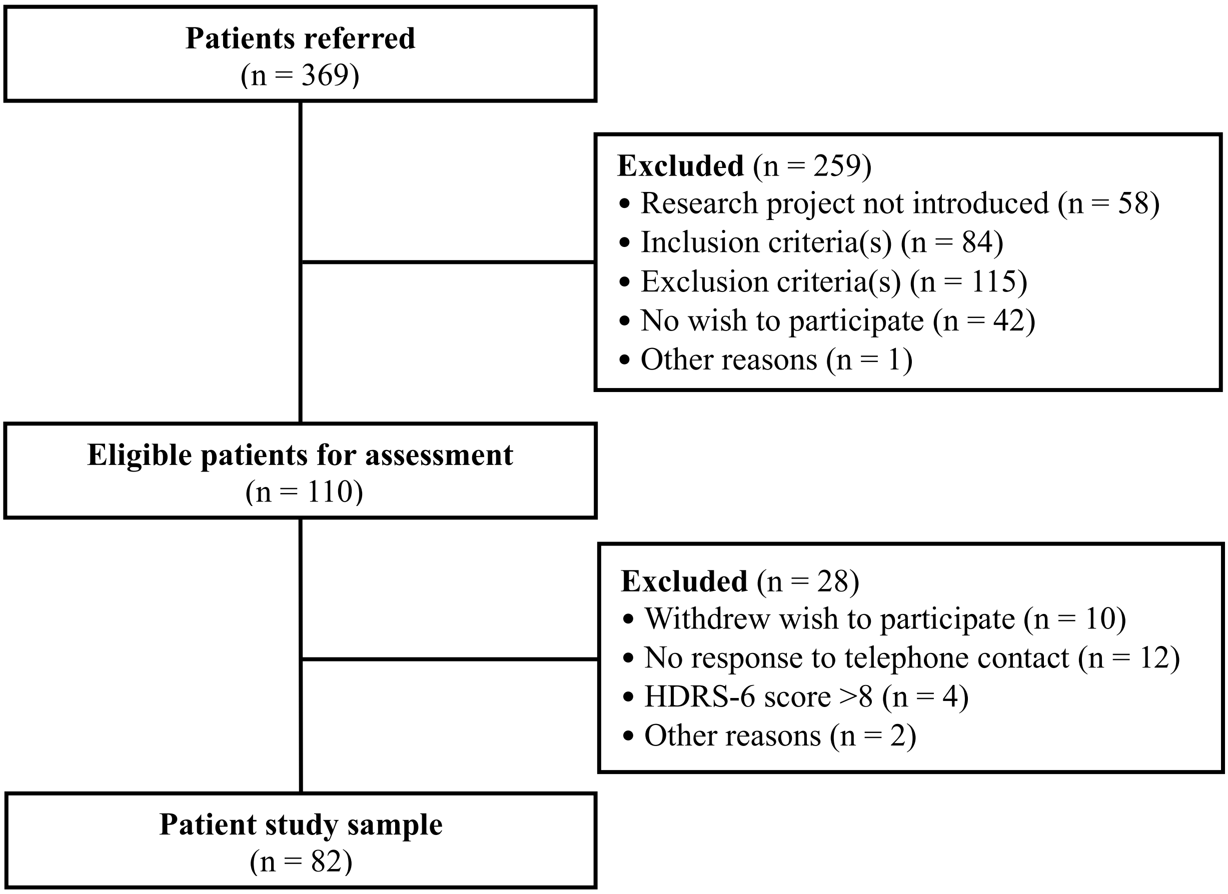

Supplementary material 3. Raw scores for the measures of objective cognitive status.

	**Patient sample (N=82)**		**HC sample (N=78,000)**	
	Mean	SD	Mean	SD
SCIP-D form 3				
Total score	74.2	8.4	77.7	8.4
VLT-I	22.5	3.0	23.2	3.0
WMT	19.0	2.4	19.9	2.4
VFT	14.9	4.2	15.5	4.2
VLT-D	7.4	2.2	7.5	2.2
PST	10.4	2.1	11.8	2.1
Standardized neuropsychological tests				
RAVLT total recall across the five learning trials (I–V)	53.0	9.0	52.6	9.4
RAVLT recall following interference (trial VI)	10.6	3.5	11.3	2.6
RAVLT recall following 30 min. delay	10.0	3.8	10.7	3.6
RBANS Digit Span Forward	8.7	2.0	9.6	1.8
WAIS-III Letter-Number Sequencing	10.0	2.2	11.3	2.0
Verbal fluency with the letters S and D	24.2	7.6	25.9	5.8
Trail Making Test part A	36.5	15.9	28.0	7.9

SCIP-D= Danish version of the Screen for Cognitive Impairment in Psychiatry; VLT-I= Verbal Learning Test, Immediate; WMT= Working Memory Test; VFT= Verbal Fluency Test; VLT-D= Verbal Learning Test, Delayed; PST= Processing Speed Test; RAVLT= Rey Auditory Verbal Learning Test; RBANS= Repeatable Battery for the Assessment of Neuropsychological Status; WAIS-III=Weschler's Adult Intelligence Scale, third version.
